# Association of surgical resection with survival in retroperitoneal leiomyosarcoma based on SEER propensity score matching and machine-learning models

**DOI:** 10.1038/s41598-026-42442-x

**Published:** 2026-03-05

**Authors:** Kun Huang, Zhenghong Huang, Yunshen He, Pan Zhao, Xiaofeng Hu

**Affiliations:** 1 Department of General Surgery, Mian Yang Hospital of Traditional Chinese Medicine, Mianyang, 621000 Sichuan People’s Republic of China; 2 Medical Insurance Department, Youxian District People’s Hospital, Mianyang, 621000 Sichuan People’s Republic of China; 3 Department of Nursing, The First Affiliated Hospital of Chongqing Medical University, Chongqing, 400000 People’s Republic of China

**Keywords:** Retroperitoneal leiomyosarcoma, Surgery, SEER, Propensity score matching, Random survival forest, Survival, Rare tumors, Cancer, Diseases, Medical research, Oncology, Risk factors

## Abstract

Retroperitoneal leiomyosarcoma (RLS) is a rare and aggressive subtype of soft tissue sarcoma with limited population-level evidence guiding surgical decision-making. This study aimed to assess the prognostic value of surgery in patients with RLS using a large real-world cohort and advanced analytical methods. Patients diagnosed with RLS between 2000 and 2019 were identified from the Surveillance, Epidemiology, and End Results (SEER) database. Propensity score matching (PSM) was used to balance baseline variables. Overall survival (OS) and cancer-specific survival (CSS) were analyzed using Kaplan–Meier curves and Cox proportional hazards models. Random survival forests (RSF) were applied to evaluate variable importance and model robustness. A total of 1041 patients were included, of whom 817 (78.5%) underwent surgery. Before matching, significant imbalances were observed in age, grade, and SEER stage. After 1:1 PSM (159 matched pairs), covariate balance was substantially improved. Surgery was associated with significantly improved survival (OS: HR = 0.34, 95% CI: 0.26–0.45; CSS: HR = 0.34, 95% CI: 0.25–0.46; both *P* < 0.001). High-grade tumors and advanced SEER stage remained independent adverse prognostic factors. RSF consistently ranked surgery, stage, and grade as the most important predictors of survival. Surgical resection status was strongly associated with survival in SEER-based analyses, but this association is subject to substantial unmeasured confounding by resectability, anatomic extent, and patient fitness; therefore, results should be interpreted as prognostic rather than causal and highlight the need for multidisciplinary assessment in high-volume sarcoma centers.

## Introduction

Retroperitoneal sarcomas (RPS) are rare, heterogeneous malignant mesenchymal tumors that account for a minority of soft-tissue sarcomas yet carry substantial morbidity and mortality due to their deep anatomic location and large size at presentation^1^. Within this group, retroperitoneal leiomyosarcoma (RLS) constitutes an aggressive histologic subtype. Histology-based analyses demonstrate that recurrence patterns and risk profiles differ markedly across RPS subtypes; in particular, leiomyosarcoma exhibits a greater propensity for distant spread than liposarcoma, underscoring the need for subtype-informed treatment strategies and robust prognostic frameworks in RLS^2^. Surgical resection remains the cornerstone of potentially curative therapy for RPS. Contemporary consensus statements emphasize management in experienced sarcoma centers with multidisciplinary planning to maximize the likelihood of complete (R0/R1) resection, guide the approach to recurrent disease, and standardize follow-up^3^. Nonetheless, optimal integration of adjunctive therapies remains unsettled. The randomized STRASS trial showed no overall improvement in abdominal recurrence-free survival with the addition of preoperative radiotherapy to surgery for primary RPS, while post-hoc and histology-specific signals continue to be debated^4^. In parallel, evidence supporting routine perioperative chemotherapy is heterogeneous and limited for most retroperitoneal histologies. Observational series focused on RLS suggest that carefully selected salvage surgery for recurrence may still yield meaningful benefit, but high-quality, subtype-specific evidence remains scarce^5^. Recent multidisciplinary reviews similarly call for large, methodologically rigorous analyses that account for selection bias and histologic diversity^6^. Population-based registries such as the Surveillance, Epidemiology, and End Results (SEER) program enable the study of rare cancers at scale by capturing detailed demographic, pathologic, treatment, and outcome data across diverse real-world settings^7–9^. Leveraging SEER can overcome many limitations of single-center experiences in RLS, where small cohorts and heterogeneity impede precise estimation of treatment effects and reliable identification of prognostic factors. However, the observational nature of registry data necessitates analytic strategies that mitigate confounding and strengthen causal interpretation.

To this end, propensity score matching (PSM) can reduce measured treatment-selection bias by balancing baseline covariates between surgical and non-surgical cohorts, with standardized differences providing recommended balance diagnostics^10^.

Complementing confounding adjustment, machine-learning-based survival modeling—particularly random survival forests (RSF)—can flexibly capture nonlinear effects and higher-order interactions and provides variable-importance rankings that summarize prognostic patterns in large registries. Importantly, however, registry-based comparisons of surgery versus no surgery are inherently susceptible to selection bias because resectability, anatomical tumor relationships, operative risk, and performance status strongly influence whether surgery is offered and are not captured in SEER. Accordingly, using SEER data (2000–2019), we examined the association between surgical resection status and overall survival (OS) and cancer-specific survival (CSS) in retroperitoneal leiomyosarcoma, and we evaluated the relative importance of routinely recorded demographic, stage, grade, and treatment variables using multivariable Cox regression and RSF-based importance analyses, while emphasizing that these methods adjust for measured confounders only and do not provide causal estimates of surgical benefit.

## Materials and methods

### Data source and study population

We conducted a population-based retrospective cohort study using the Surveillance, Epidemiology, and End Results (SEER) program. Cases with a pathological diagnosis of RLS from January 1, 2000, to December 31, 2019, were identified. Data were retrieved with SEER*Stat (v8.3.9). SEER data are de-identified and publicly available; therefore, this study was exempt from institutional review board oversight and informed consent requirements.

### Case identification, inclusion, and exclusion criteria

RLS was defined using ICD-O-3 histology codes for leiomyosarcoma (8890/3, 8891/3, 8896/3) and a retroperitoneum primary site. Inclusion criteria were: (1) primary RLS at initial diagnosis; (2) pathologically confirmed; (3) diagnosis between 2000–2019; and (4) available survival data.

Exclusion criteria were: (1) cases identified by autopsy or death certificate only; (2) multiple primary tumors with RLS not the first malignancy; and (3) missing or incomplete key variables required for analysis (e.g., survival time, vital status, surgical status, stage, or grade).

### Variables and definitions

From SEER, we extracted demographics (age at diagnosis, sex, race), tumor characteristics (tumor differentiation as recorded in the SEER grade/differentiation field), SEER summary stage (localized, regional, distant, unknown), treatment variables (surgery, radiotherapy, chemotherapy), and survival outcomes. SEER summary stage was analyzed using the SEER Summary Stage system, in which “localized” indicates disease confined to the site of origin, “regional” generally reflects contiguous/direct extension into adjacent tissues and/or organs (and/or regional lymph nodes), and “distant” denotes metastatic disease. Given that lymph node metastases are uncommon in retroperitoneal leiomyosarcoma, SEER “regional” stage in this context most often represents locally advanced tumors with direct extension rather than nodal spread; therefore, we interpreted this category accordingly and note that SEER stage categories are registry constructs that may not map directly onto specialist retroperitoneal sarcoma terminology.

- *Age* was dichotomized at 60 years (≤ 60 vs > 60) based on prior literature and clinical relevance.
- *Tumor differentiation (SEER grade/differentiation field)* was grouped as low differentiation grade (I/II: well/moderately differentiated), high differentiation grade (III/IV: poorly differentiated and undifferentiated/anaplastic; SEER grade IV), or unknown. This SEER variable reflects tumor differentiation and is not equivalent to a dedicated sarcoma grading system (e.g., the French Federation of Cancer Centers Sarcoma Group (FNCLCC) grading system).
- *Race* was categorized as White, Black, or Other (including American Indian/Alaska Native and Asian/Pacific Islander).
- *Treatment variables* (surgery, radiotherapy, chemotherapy) were extracted from SEER treatment fields and categorized as **yes, no, or unknown** (if applicable), reflecting treatments delivered during the initial course of therapy for the primary tumor episode.
- *Survival outcomes* included overall survival (OS) and cancer-specific survival (CSS). OS was defined as time from diagnosis to death from any cause, and CSS as time from diagnosis to death attributed to the index cancer, with patients censored at last follow-up if alive (or if death occurred from other causes for CSS).

### Endpoints

Primary endpoints were OS and CSS:

- OS was defined as time (months) from diagnosis to death from any cause; patients alive at last follow-up were censored.
- CSS was defined as the time from diagnosis to RLS-specific death; deaths from other causes and survivors at last follow-up were censored. The administrative follow-up cutoff in SEER for this extraction was December 31, 2019.

### Propensity score matching (PSM)

Because treatment allocation (surgery vs no surgery) is non-random in observational data, we implemented 1:1 nearest-neighbor PSM without replacement on the logit of the propensity score to balance baseline characteristics between surgical and non-surgical cohorts. The propensity model included age group, sex, race, SEER stage, differentiation grade, radiotherapy, and chemotherapy. A caliper of 0.01 was used to restrict matches. Covariate balance after matching was assessed using absolute standardized mean differences (SMDs), with SMD ≤ 0.10 considered acceptable balance^10^. However, propensity score methods can only adjust for measured covariates; unmeasured confounding—particularly factors related to technical resectability, anatomic tumor extent, patient fitness, and center expertise that are not captured in SEER—may still remain.

### Survival analysis and multivariable modeling

Kaplan–Meier methods were used to estimate OS and CSS, with log-rank tests comparing survival curves between groups. We fitted Cox proportional hazards models to estimate hazard ratios (HRs) and 95% confidence intervals (CIs). Variables with clinical relevance and/or *P* < 0.10 in univariable analyses were entered into multivariable models. Variance inflation factors (VIFs) were examined to assess multicollinearity; VIF < 10 was considered acceptable. Proportional hazards assumptions were verified using Schoenfeld residuals and log–log plots.

### Machine-learning survival modeling

To complement regression modeling and rank prognostic importance without parametric assumptions, we trained RSF models on the matched cohort. Candidate predictors included all covariates above, plus treatment (surgery). We used standard hyperparameters (e.g., number of trees ≥ 1000; log-rank splitting; internal out-of-bag estimation) and computed variable importance scores to identify the strongest predictors of OS and CSS^11,12^.

### Software

All analyses were performed with Stata/MP 16.0 (StataCorp) and R 4.2.3 (R Foundation for Statistical Computing). PSM was implemented with standard matching routines; RSF modeling used established survival-tree packages. A two-sided α = 0.05 defined statistical significance.

### Ethics statement

This study used de-identified SEER data and complied with the Declaration of Helsinki. No human subjects were directly involved; IRB approval and informed consent were not required.

## Results

### Baseline characteristics before and after matching

A total of 1041 patients with RLS met the inclusion criteria, including 817 (78.5%) treated with surgery and 224 (21.5%) without surgery. Baseline clinicopathological characteristics were imbalanced between groups before matching (*P* < 0.05 for multiple covariates). After 1:1 propensity score matching (PSM), 318 patients were retained (159 per group) with well-balanced baseline features (*P* > 0.05 across covariates). The median follow-up was 17 months (IQR, 6–41) (Table 1).

**Table 1:** Baseline characteristics of patients with retroperitoneal leiomyosarcoma (RLS) before and after propensity score matching (PSM).

Variable	Before matching				After matching			
	All (n = 1,041)	No surgery (n = 224)	Surgery (n = 817)	*P*	All (n = 318)	No surgery (n = 159)	Surgery (n = 159)	*P*
Age, n (%)				< 0.001				0.084
≤ 60 years	494 (47.5)	76 (33.9)	418 (51.2)		93 (29.2)	54 (34.0)	39 (24.5)	
> 60 years	547 (52.5)	148 (66.1)	399 (48.8)		225 (70.8)	105 (66.0)	120 (75.5)	
Race, n (%)				0.031				0.952
White	732 (70.3)	146 (65.2)	586 (71.7)		221 (69.5)	110 (69.2)	111 (69.8)	
Black	201 (19.3)	57 (25.4)	144 (17.6)		68 (21.4)	35 (22.0)	33 (20.8)	
Other	108 (10.4)	21 (9.4)	87 (10.6)		29 (9.1)	14 (8.8)	15 (9.4)	
Sex, n (%)				0.011				0.525
Male	277 (26.6)	75 (33.5)	202 (24.7)		84 (26.4)	45 (28.3)	39 (24.5)	
Female	764 (73.4)	149 (66.5)	615 (75.3)		234 (73.6)	114 (71.7)	120 (75.5)	
Differentiation grade, n (%)				< 0.001				0.853
I/II	252 (24.2)	22 (9.8)	230 (28.2)		43 (13.5)	21 (13.2)	22 (13.8)	
III/IV	438 (42.1)	71 (31.7)	367 (44.9)		114 (35.8)	55 (34.6)	59 (37.1)	
Unknown	351 (33.7)	131 (58.5)	220 (26.9)		161 (50.6)	83 (52.2)	78 (49.1)	
SEER summary stage, n (%)				< 0.001				0.351
Localized	305 (29.3)	28 (12.5)	277 (33.9)		61 (19.2)	25 (15.7)	36 (22.6)	
Regional	301 (28.9)	48 (21.4)	253 (31.0)		82 (25.8)	41 (25.8)	41 (25.8)	
Distant	180 (17.3)	79 (35.3)	101 (12.4)		78 (24.5)	44 (27.7)	34 (21.4)	
Unknown	255 (24.5)	69 (30.8)	186 (22.8)		97 (30.5)	49 (30.8)	48 (30.2)	
Radiotherapy, n (%)				< 0.001				0.137
No	694 (66.7)	176 (78.6)	518 (63.4)		227 (71.4)	120 (75.5)	107 (67.3)	
Yes	347 (33.3)	48 (21.4)	299 (36.6)		91 (28.6)	39 (24.5)	52 (32.7)	
Chemotherapy, n (%)				< 0.001				0.820
No	737 (70.8)	107 (47.8)	630 (77.1)		187 (58.8)	95 (59.7)	92 (57.9)	
Yes	304 (29.2)	117 (52.2)	187 (22.9)		131 (41.2)	64 (40.3)	67 (42.1)	

SEER, Surveillance, Epidemiology, and End Results; PSM, propensity score matching.

*P*-values from χ^2^ tests comparing surgery vs no surgery within each period (before or after matching). Percentages may not total 100% due to rounding.

### Survival outcomes in the matched cohort

**Fig. 1:**
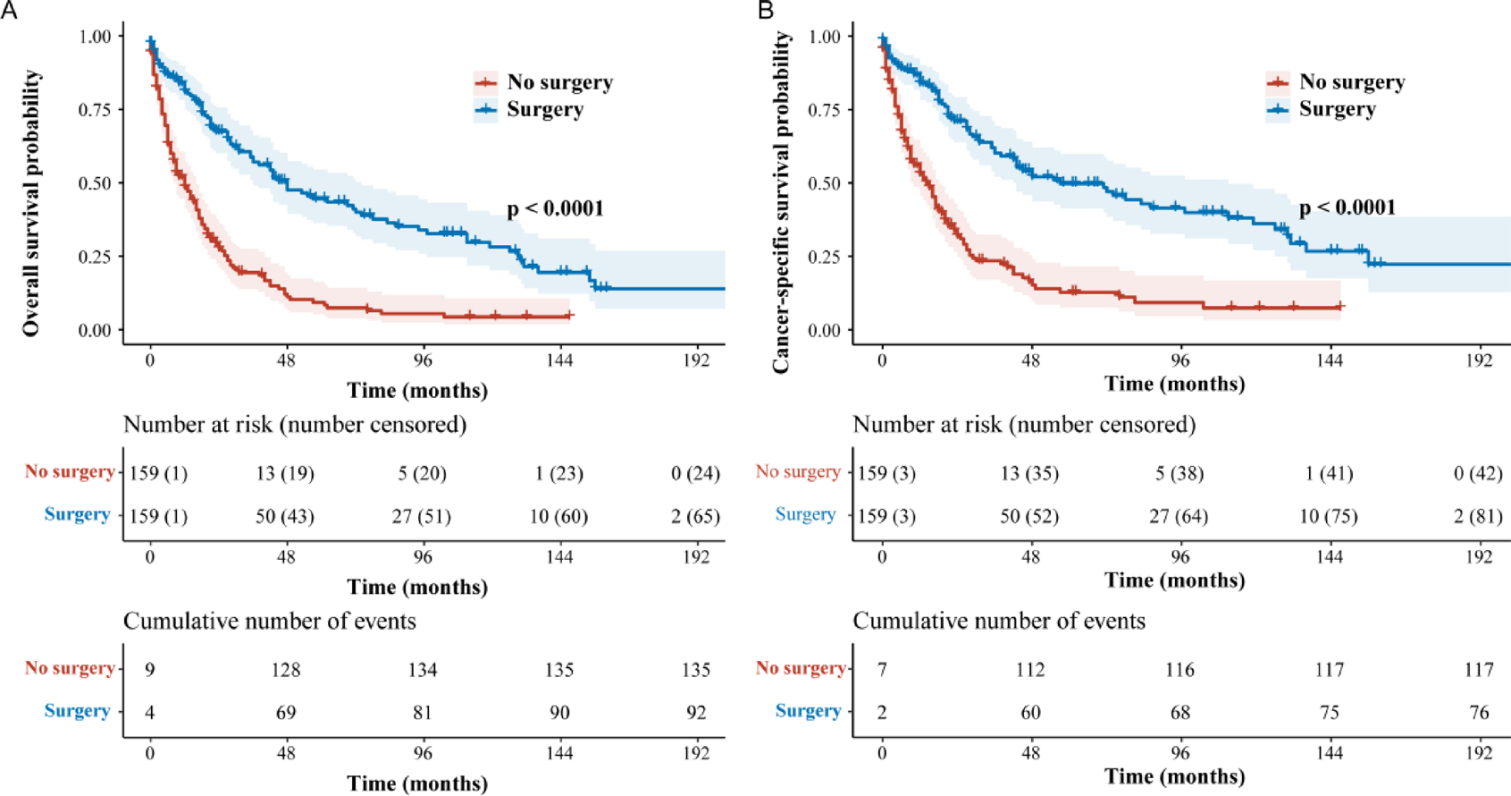
Kaplan–Meier survival by surgery after propensity score matching. (**A**) Overall survival (OS) and (**B**) cancer-specific survival (CSS) in the matched cohort (n = 318; surgery n = 159, no surgery n = 159). Curves compare surgery (blue) versus no surgery (red) with 95% confidence-interval ribbons; tick marks denote censoring. Risk tables display the number at risk (number censored) at prespecified time points, with cumulative numbers of events reported below. Group differences by log-rank test were significant for both endpoints (*P* < 0.001). Abbreviations: OS, overall survival; CSS, cancer-specific survival.

Within the matched cohort, 92/159 (57.9%) patients in the surgery group died, including 76 (47.8%) cancer-specific deaths; corresponding figures in the non-surgery group were 135/159 (84.9%) and 117 (73.6%), respectively. One-year OS was 83.14% with surgery versus 51.46% without surgery, and one-year CSS was 85.03% versus 55.58%, respectively (all *P* < 0.001 by log-rank). Reported log-rank statistics were χ^2^ = 60.15 for OS and χ^2^ = 52.00 for CSS (both *P* < 0.001) (Fig. 1).

### Univariable cox analysis

On univariable analysis, tumor stage, differentiation grade, surgery, and radiotherapy were associated with both OS and CSS; poorer differentiation and more advanced stage predicted worse outcomes, while surgery and radiotherapy showed protective associations (all *P* < 0.05). Age, sex, and race were not significant in univariable models for both endpoints (Table 2).

**Table 2:** Univariable Cox proportional hazards analysis for overall survival (OS) and cancer-specific survival (CSS).

Variable	OS HR (95% CI)	*P*	CSS HR (95% CI)	*P*
*Age (years)*				
≤ 60 (ref)	1 (ref)	—	1 (ref)	—
> 60	1.07 (0.81–1.43)	0.622	0.92 (0.68–1.25)	0.605
*Race*				
White (ref)	1 (ref)	—	1 (ref)	—
Black	1.01 (0.73–1.39)	0.943	1.01 (0.71–1.43)	0.972
Other	0.85 (0.53–1.36)	0.491	0.95 (0.58–1.55)	0.834
*Sex*				
Male (ref)	1 (ref)	—	1 (ref)	—
Female	0.90 (0.67–1.22)	0.501	0.86 (0.62–1.19)	0.363
*Differentiation grade*				
I/II (ref)	1 (ref)	—	1 (ref)	—
III/IV	1.46 (0.97–2.19)	0.068	1.71 (1.09–2.70)	0.020
Unknown	1.32 (0.88–1.98)	0.173	1.39 (0.88–2.19)	0.157
*SEER summary stage*				
Localized (ref)	1 (ref)	—	1 (ref)	—
Regional	1.41 (0.97–2.07)	0.075	1.48 (0.96–2.28)	0.074
Distant	2.75 (1.87–4.04)	< 0.001	3.20 (2.09–4.89)	< 0.001
Unknown	1.30 (0.82–2.05)	0.259	1.42 (0.86–2.34)	0.169
*Radiotherapy*				
No (ref)	1 (ref)	—	1 (ref)	—
Yes	0.70 (0.52–0.93)	0.014	0.73 (0.54–1.00)	0.047
*Chemotherapy*				
No (ref)	1 (ref)	—	1 (ref)	—
Yes	1.02 (0.78–1.33)	0.892	1.14 (0.86–1.52)	0.373
*Surgery*				
No (ref)	1 (ref)	—	1 (ref)	—
Yes	0.34 (0.26–0.45)	< 0.001	0.34 (0.25–0.46)	< 0.001

CI, confidence interval; SEER, Surveillance, Epidemiology, and End Results.

### Multivariable cox analysis

**Fig. 2:**
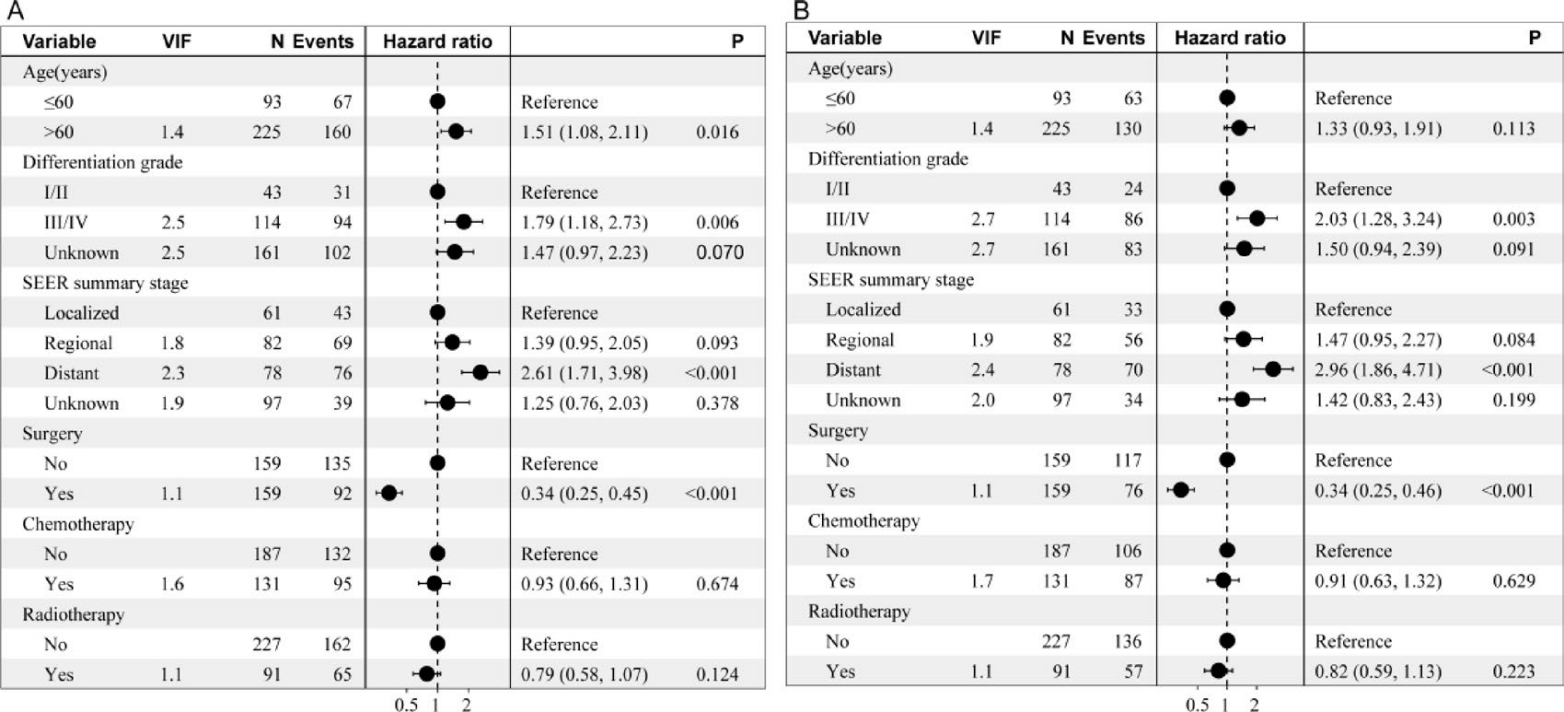
Multivariable Cox models for survival after propensity score matching. Forest plots display adjusted hazard ratios (HRs) with 95% confidence intervals for (**A**) OS and (**B**) CSS. Covariates included age (> 60 vs ≤ 60 years), sex (female vs male), race (Black/Other vs White), differentiation grade (III/IV or Unknown vs I/II), SEER summary stage (Regional/Distant/Unknown vs Localized), radiotherapy (yes vs no), chemotherapy (yes vs no), and surgery (yes vs no). HR < 1 favors the category listed second in each contrast (e.g., surgery). Proportional-hazards assumptions were evaluated using Schoenfeld residuals; two-sided α = 0.05. HR, hazard ratio; CI, confidence interval; SEER, Surveillance, Epidemiology, and End Results.

In multivariable models, age, tumor stage, differentiation grade, and surgery were independent predictors of OS (all *P* < 0.05), whereas tumor stage, differentiation grade, and surgery independently predicted CSS (all *P* < 0.05). Receipt of surgery remained strongly associated with improved outcomes in the matched cohort (OS HR = 0.34, 95% CI 0.25–0.45; CSS HR = 0.34, 95% CI 0.25–0.46; both *P* < 0.001) (Fig. 2), noting that this association may also reflect underlying selection related to resectability and patient fitness.

### Machine-learning survival modeling

**Fig. 3:**
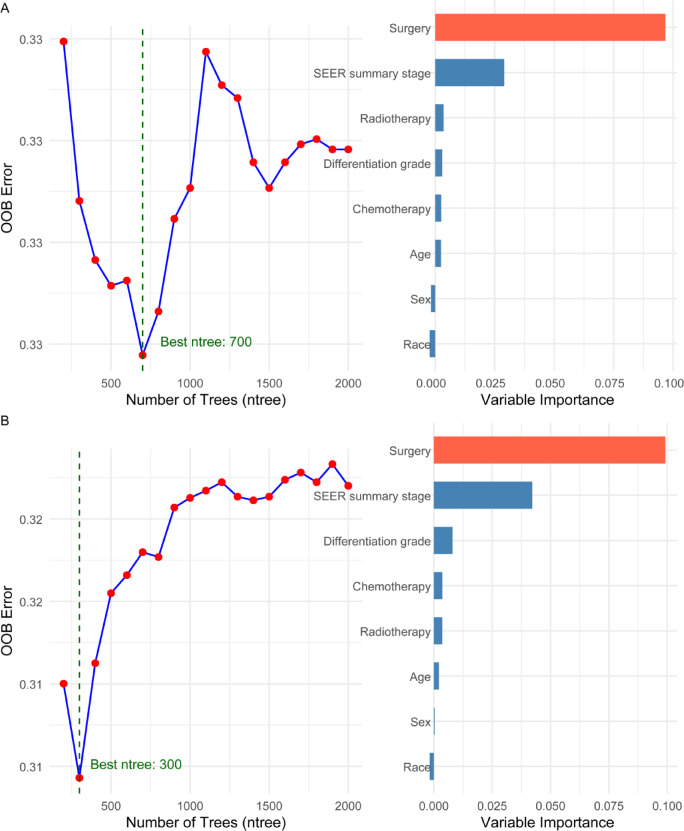
Random survival forest (RSF) tuning and variable importance. For (**A**) OS and (**B**) CSS, the left panels plot out-of-bag (OOB) error against the number of trees; the vertical dashed line marks the ntree with the lowest OOB error (best ntree). Right panels show permutation-based variable importance ranked from highest to lowest; larger bars indicate greater predictive contribution, with surgery, SEER summary stage, and differentiation grade among the top predictors. RSF models used log-rank splitting and internal OOB estimation; details appear in Methods. RSF, random survival forest; OOB, out-of-bag; SEER, Surveillance, Epidemiology, and End Results.

RSF analysis corroborated regression findings: among all candidate variables, the highest importance scores for both OS and CSS were observed for surgery, followed by tumor stage and differentiation grade (Fig. 3).

## Discussion

In this population-based analysis of retroperitoneal leiomyosarcoma, receipt of surgical resection was strongly associated with longer overall and cancer-specific survival after propensity score matching, while SEER summary stage and tumor differentiation remained independently associated with outcomes. Concordant results from propensity-adjusted Cox models and random survival forests suggested a stable ordering of prognostic features, with surgery status, stage, and differentiation consistently ranked among the most influential predictors in our models^10,11^. These findings are broadly aligned with contemporary recommendations that emphasize complete gross resection as the cornerstone of curative-intent management for retroperitoneal sarcomas when feasible and delivered within multidisciplinary teams at experienced centers^13–15^. Evidence from randomized trials, including the EORTC STRASS study, did not demonstrate an overall benefit of routine preoperative radiotherapy across histologies, which is consistent with our observation that radiotherapy was not among the top prognostic features in this registry-based analysis^4^. Prior histology-aware studies have highlighted leiomyosarcoma’s propensity for distant relapse and the role of surgery in selected settings, supporting risk-adapted surveillance and management strategies^2,5^. Importantly, SEER reflects care delivered across a wide spectrum of institutions, including many low-volume centers, and outcomes may differ meaningfully by center volume and multidisciplinary expertise^16,17^. In contrast, high-volume reference-center series from Trans-Atlantic Retroperitoneal Sarcoma Working Group represent outcomes achieved within specialized sarcoma pathways. Accordingly, the absolute survival estimates observed in SEER should be interpreted as population-level real-world outcomes and may not be directly comparable to results from high-volume sarcoma centers; nonetheless, both settings underscore the importance of expert multidisciplinary evaluation and appropriate patient selection.

A central consideration in interpreting these results is the profound selection inherent to surgical management of retroperitoneal sarcoma. In routine practice, the decision to operate is determined by nuanced assessments of anatomical relationships and technical resectability (e.g., involvement of the retrohepatic inferior vena cava, hepatic veins, portal structures, aorta, and renal hilum/vasculature), as well as patient fitness and anticipated perioperative risk. Consequently, patients recorded as not undergoing surgery in SEER are frequently those deemed unresectable or unsafe to resect, rather than patients for whom curative-intent surgery was appropriate but omitted. Because SEER lacks granular data on tumor complexity, resectability, margin status, operative intent, perioperative morbidity, and center expertise, neither propensity score matching nor machine-learning models can fully adjust for these determinants. Therefore, the observed survival differences should be interpreted as prognostic associations rather than evidence that surgery would be beneficial or appropriate for all patients, and they underscore the importance of multidisciplinary resectability assessment in specialized sarcoma centers.

Methodologically, pairing propensity score matching with random survival forests strengthens inference and clinical interpretability. Propensity techniques—with standardized balance diagnostics—address measured selection bias and enable fairer comparisons between surgical and non-surgical cohorts^10^. Random survival forests, free of proportional-hazards and linearity assumptions, capture nonlinearities and interactions while providing variable-importance rankings that are intuitive for clinicians; in our matched data, these rankings reproduced the regression hierarchy and amplified confidence in the primacy of surgery and the roles of stage and grade^6,11,15^.

This work has important limitations that are particularly relevant to interpreting surgery in retroperitoneal leiomyosarcoma. Most critically, SEER does not capture technical resectability, detailed anatomical extent (including major vascular/organ involvement), performance status, perioperative risk, surgeon judgment, or center expertise—factors that strongly determine whether surgery is offered and cannot be fully addressed by propensity score methods or machine-learning models. Consequently, residual confounding and selection bias are expected, and the observed associations should not be construed as causal. In addition, registry data lack surgical margin status, tumor size/volume and complexity, vascular encasement, detailed treatment intent and sequencing, perioperative morbidity, recurrence patterns, and center-level quality metrics, which limits clinical granularity, increases heterogeneity, and may influence both observed survival and treatment-selection patterns^10,11^. Despite these limitations, the results provide population-level context and motivate clearer next steps. Practice patterns and perioperative strategies continue to evolve under guideline influence, and histology-tailored systemic approaches remain under active evaluation^13,14^. Prospective, leiomyosarcoma-focused trials—such as STRASS2, which evaluates neoadjuvant chemotherapy versus surgery alone—may better define which patients are most likely to benefit from systemic therapy in addition to surgery, and systematic capture of tumor biology and biomarkers is needed to address the persistent risk of distant relapse^18,19^. Finally, integrating centralization with measurable quality and multidisciplinary-process metrics may help translate population-level insights into more consistent outcomes across care settings^16^.

## Conclusions

In this population-based SEER analysis, receipt of surgery was strongly associated with survival and emerged as a leading prognostic factor across both conventional regression and machine-learning survival models. However, because SEER lacks key determinants of surgical candidacy—especially technical resectability, anatomical tumor complexity, margin feasibility, operative risk, and performance status—these findings should not be interpreted as evidence that surgery is appropriate or beneficial for all patients. Clinical decision-making should remain individualized within multidisciplinary sarcoma teams, and our results primarily underscore the importance of timely referral for expert assessment of resectability and comprehensive sarcoma care.

## Data Availability

The study analyzed de-identified, publicly available data from the SEER Program (SEER*Stat version 8.3.9). Researchers can obtain access via the SEER website by completing the standard data-use agreement. The analytic code and variable definitions used in this study are available from the corresponding author upon reasonable request.

## References

[1] Porter, G. A., Baxter, N. N. & Pisters, P. W. Retroperitoneal sarcoma: A population-based analysis of epidemiology, surgery, and radiotherapy. *Cancer* **106**(7), 1610–1616. 10.1002/cncr.21761 (2006). 16518798 10.1002/cncr.21761

[2] Tan, M. C. et al. Histology-based classification predicts pattern of recurrence and improves risk stratification in primary retroperitoneal sarcoma. *Ann. Surg.* **263**(3), 593–600. 10.1097/sla.0000000000001149 (2016). 25915910 10.1097/SLA.0000000000001149 PMC4619189

[3] Tseng, W. W. et al. Management of locally recurrent retroperitoneal sarcoma in the adult: An updated consensus approach from the Transatlantic Australasian Retroperitoneal Sarcoma Working Group. *Ann. Surg. Oncol.* **29**(12), 7335–7348. 10.1245/s10434-022-11864-y (2022). 35767103 10.1245/s10434-022-11864-y

[4] Bonvalot, S. et al. Preoperative radiotherapy plus surgery versus surgery alone for patients with primary retroperitoneal sarcoma (EORTC-62092: STRASS): A multicentre, open-label, randomised, phase 3 trial. *Lancet Oncol.* **21**(10), 1366–1377. 10.1016/s1470-2045(20)30446-0 (2020). 32941794 10.1016/S1470-2045(20)30446-0

[5] Ikoma, N. et al. Recurrence patterns of retroperitoneal leiomyosarcoma and impact of salvage surgery. *J. Surg. Oncol.* **116**(3), 313–319. 10.1002/jso.24667 (2017). 28557016 10.1002/jso.24667 PMC5937256

[6] De Bree, E. et al. Retroperitoneal soft tissue sarcoma: Emerging therapeutic strategies. *Cancers (Basel)*10.3390/cancers15225469 (2023). 38001729 10.3390/cancers15225469 PMC10670057

[7] Duggan, M. A. et al. The Surveillance, Epidemiology, and End Results (SEER) Program and pathology: Toward strengthening the critical relationship. *Am. J. Surg. Pathol.* **40**(12), e94–e102. 10.1097/pas.0000000000000749 (2016). 27740970 10.1097/PAS.0000000000000749 PMC5106320

[8] Friedman, S. & Negoita, S. History of the Surveillance, Epidemiology, and End Results (SEER) Program. *JNCI Monogr.* **2024**(65), 105–109. 10.1093/jncimonographs/lgae033 (2024). 10.1093/jncimonographs/lgae033 PMC11300016 39102881

[9] Penberthy, L. & Friedman, S. The SEER Program’s evolution: Supporting clinically meaningful population-level research. *JNCI Monogr.* **2024**(65), 110–117. 10.1093/jncimonographs/lgae022 (2024). 10.1093/jncimonographs/lgae022 PMC11300003 39102886

[10] Austin, P. C. Balance diagnostics for comparing the distribution of baseline covariates between treatment groups in propensity-score matched samples. *Stat. Med.* **28**(25), 3083–3107. 10.1002/sim.3697 (2009). 19757444 10.1002/sim.3697 PMC3472075

[11] Ishwaran, H. et al. Random survival forests for competing risks. *Biostatistics (Oxford, England)* **15**(4), 757–773. 10.1093/biostatistics/kxu010 (2014). 24728979 10.1093/biostatistics/kxu010 PMC4173102

[12] Wang, H. & Li, G. A selective review on random survival forests for high dimensional data. *Quant. Bio-Sci.* **36**(2), 85–96. 10.22283/qbs.2017.36.2.85 (2017). 10.22283/qbs.2017.36.2.85 PMC6364686 30740388

[13] Gronchi, A. et al. Soft tissue and visceral sarcomas: ESMO-EURACAN-GENTURIS clinical practice guidelines for diagnosis, treatment and follow-up(☆). *Ann. Oncol.* **32**(11), 1348–1365. 10.1016/j.annonc.2021.07.006 (2021). 34303806 10.1016/j.annonc.2021.07.006

[14] Von Mehren, M. et al. Soft tissue sarcoma, version 2.2022, NCCN Clinical Practice Guidelines in Oncology. *J. Natl. Compr. Canc. Netw.* **20**(7), 815–833. 10.6004/jnccn.2022.0035 (2022). 35830886 10.6004/jnccn.2022.0035 PMC10186762

[15] Swallow, C. J. et al. Management of primary retroperitoneal sarcoma (RPS) in the adult: An updated consensus approach from the Transatlantic Australasian RPS Working Group. *Ann. Surg. Oncol.* **28**(12), 7873–7888. 10.1245/s10434-021-09654-z (2021). 33852100 10.1245/s10434-021-09654-z PMC9257997

[16] Kamarajah, S. K. et al. Association between centre volume and allocation to curative surgery and long-term survival for retroperitoneal sarcoma. *BJS Open.*10.1093/bjsopen/zrad059 (2023). 37498965 10.1093/bjsopen/zrad059 PMC10373904

[17] Stillman, M., Espat, N. J. & Kwon, S. Validation of updated sarculator nomogram for primary retroperitoneal Sarcoma in the United States. *Ann Surg Oncol*10.1245/s10434-025-18873-7 (2025). 41369816 10.1245/s10434-025-18873-7

[18] Lambdin, J. et al. A randomized phase III study of neoadjuvant chemotherapy followed by surgery versus surgery alone for patients with high-risk retroperitoneal sarcoma (STRASS2). *Ann. Surg. Oncol.* **30**(8), 4573–4575. 10.1245/s10434-023-13500-9 (2023). 37170037 10.1245/s10434-023-13500-9 PMC10687743

[19] Øines, M. N. et al. Leiomyosarcoma of the abdomen and retroperitoneum; a systematic review. *Front. Surg.* **11**, 1375483. 10.3389/fsurg.2024.1375483 (2024). 39086921 10.3389/fsurg.2024.1375483 PMC11288885

